# HLA-B*44 and C*01 Prevalence Correlates with Covid19 Spreading across Italy

**DOI:** 10.3390/ijms21155205

**Published:** 2020-07-23

**Authors:** Pierpaolo Correale, Luciano Mutti, Francesca Pentimalli, Giovanni Baglio, Rita Emilena Saladino, Pierpaolo Sileri, Antonio Giordano

**Affiliations:** 1 Unit of Medical Oncology, Oncology Department, Grand Metropolitan Hospital ‘Bianchi Melacrino Morelli’, I-89124 Reggio Calabria, Italy; correalep@yahoo.it; 2 Sbarro Institute for Cancer Research and Molecular Medicine, Center for Biotechnology, College of Science and Technology, Temple University, Philadelphia, PA 19122, USA; luciano.mutti@hotmail.it; 3 Cell Biology and Biotherapy Unit, Istituto Nazionale Tumori, IRCCS, Fondazione G. Pascale, I-80131 Napoli, Italy; f.pentimalli@istitutotumori.na.it; 4 Ministry of Health, I-00153 Rome, Italy; g.baglio@sanita.it; 5 Tissue Typing Unit, Grand Metropolitan Hospital ‘Bianchi Melacrino Morelli’, I-89124 Reggio Calabria, Italy; ritaemilena.saladino@gmail.com; 6 Università Vita Salute San Raffaele, I-20132 Milano, Italy; Sileri.pierpaolo@hsr.it; 7 Department of Medical Biotechnologies, University of Siena, I-53100 Siena, Italy

**Keywords:** SARS-Cov2, coronavirus, COVID-19, HLA class I, viral infection susceptibility

## Abstract

The spread of COVID-19 is showing huge, unexplained, differences between northern and southern Italy. We hypothesized that the regional prevalence of specific class I human leukocyte antigen (HLA) alleles, which shape the anti-viral immune response, might partly underlie these differences. Through an ecological approach, we analyzed whether a set of HLA alleles (A, B, C), known to be involved in the immune response against infections, correlates with COVID-19 incidence. COVID-19 data were provided by the National Civil Protection Department, whereas HLA allele prevalence was retrieved through the Italian Bone-Marrow Donors Registry. Among all the alleles, HLA-A*25, B*08, B*44, B*15:01, B*51, C*01, and C*03 showed a positive log-linear correlation with COVID-19 incidence rate fixed on 9 April 2020 in proximity of the national outbreak peak (Pearson’s coefficients between 0.50 and 0.70, *p*-value < 0.0001), whereas HLA-B*14, B*18, and B*49 showed an inverse log-linear correlation (Pearson’s coefficients between −0.47 and −0.59, *p*-value < 0.0001). When alleles were examined simultaneously using a multiple regression model to control for confounding factors, HLA-B*44 and C*01 were still positively and independently associated with COVID-19: a growth rate of 16% (95%CI: 0.1–35%) per 1% point increase in B*44 prevalence; and of 19% (95%CI: 1–41%) per 1% point increase in C*01 prevalence. Our epidemiologic analysis, despite the limits of the ecological approach, is strongly suggestive of a permissive role of HLA-C*01 and B*44 towards SARS-CoV-2 infection, which warrants further investigation in case-control studies. This study opens a new potential avenue for the identification of sub-populations at risk, which could provide Health Services with a tool to define more targeted clinical management strategies and priorities in vaccination campaigns.

## 1. Introduction

**Figure 1:**
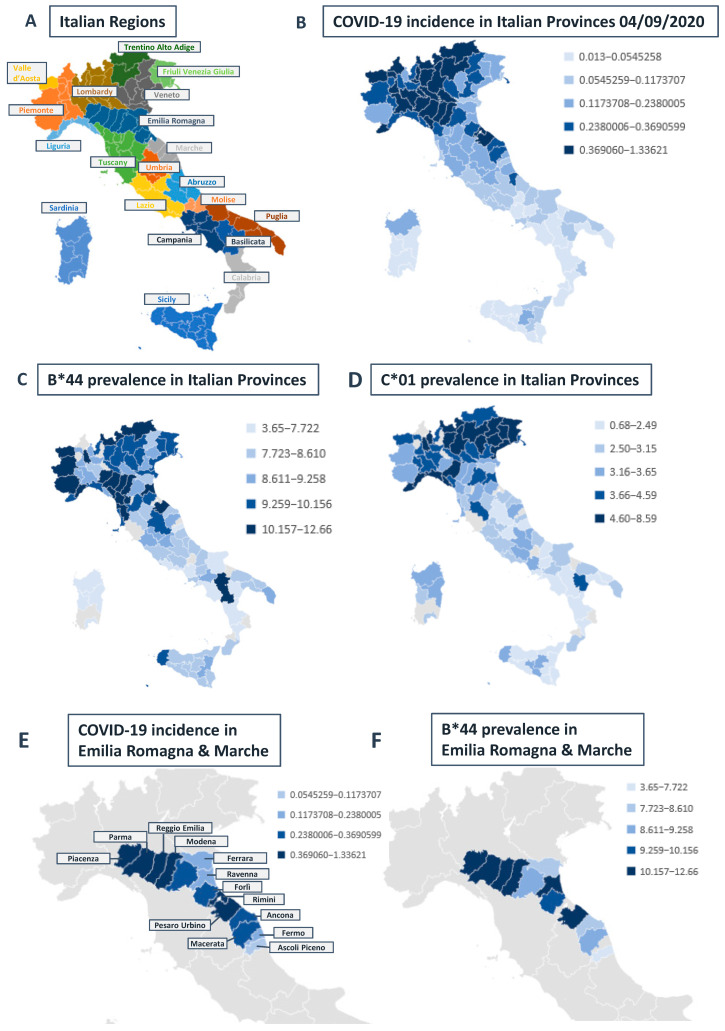
COVID-19 incidence, human leukocyte antigen (HLA)-B*44 and C*01 prevalence in Italian Provinces. (**A**): The graphical map shows the twenty Italian regions each constituted by various provinces. (**B**): The graphical map shows quintiles of COVID-19 incidence across Italian provinces. Incidence data were calculated as the number of laboratory-confirmed COVID-19 cases up to 04/09/2020 divided by the number of residents, according to the official national data (supplementary data). (**C**,**D**). The graphical maps show B*44 and C*01 prevalence (%) in Italian Provinces. (**E**,**F**) The graphical maps show COVID-19 incidence and B*44 prevalence (%) in the provinces of Emilia Romagna and Marche. Geographical maps were built through Microsoft Excel. All COVID-19 incidence and HLA prevalence values are reported as Supplementary Data.

COVID-19 has been declared a pandemic by the WHO [1]. Italy showed an explosive and apparently unrestrainable evolution throughout the country rapidly achieving one of the highest infection and mortality rates worldwide since the first case diagnosed in the province of Lodi in Lombardy, on 21 February 2020 [2]. Italian authorities are strictly monitoring the outbreak and report a large gradient of frequency that decreases from the northern to the southern and the islands, across the twenty regions of the country [3]. To date, this gradient has not been significantly modified even though the epidemic had the possibility to spread all along the peninsula due to massive migratory fluxes of individuals escaping from the high-risk regions to return in their native landscapes [4] and a delayed restrictive response by the different regional authorities until March 2020. The incidence of COVID-19 cases reported by the national authorities also indicates relevant differences in the infection spreading within single provincial areas composing some of the most affected Italian regions (Figure 1a,b). Many socio-political as well as environmental hypotheses have been proposed to explain inter- and intra-regional differences and the reasons for such an aggressive spreading throughout northern Italy but, to date, no clear demonstration has been provided.

Emerging data show that both T cell and humoral response to COVID-19 infection may equally contribute to virus clearance and protective memory [2,5], however in a minority of infected patients, an inappropriate immune-response may lead to virus spread from the oropharyngeal district to the lung and other tissues, including kidney and the central nervous system (CNS) [2,6,7]. Moreover, an exaggerated cell mediated response to the virus in the alveolar tissue may be responsible for the dreaded cytokines storm and interstitial pneumonitis, leading to a fatal acute respiratory distress syndrome (ARDS) [2,8,9,10].

Considering the crucial role played by Class I/II human leukocyte antigen (HLA) molecules in triggering the anti-viral immune-response, it has been hypothesized that different HLA alleles may define an individual susceptibility to COVID-19 infection and spreading, as reported for other viruses as well as the two different corona-viruses responsible for Severe Acute Respiratory Syndrome (SARS) and Middle East Respiratory Syndrome (MERS) [5]. In this context, several studies are searching for selected HLAs with a very efficient ability to present viral-antigen-derived epitope peptides to cytotoxic T cells. The identification of highly immunogenic peptide epitopes recognized by specific T Cell Receptors (TCRs) might, in fact, provide potential candidates for vaccine development [11,12,13]. By triggering and sustaining the human host immune-defences to the virus, specific Class I HLA alleles may also be involved in the occurrence of other symptoms, morbidity or lethality. Indeed, a study in a small cohort of COVID-19 Chinese patients suggested that specific HLA alleles might correlate with disease occurrence [14]. Therefore, we set out to perform an exploratory epidemiological analysis, through an ecological approach aiming to investigate whether known differences existing in HLA-A, B, and C allele distribution among the Italian population could be correlated to COVID-19 incidence and spread throughout the peninsula. 

## 2. Results

### Correlation between HLA-A, B, and C Allele Frequency and COVID-19 Incidence in Italian Provinces 

We extrapolated HLA-A, B, and C allele frequency within the different Italian regions and relative intraregional provinces from the Italian Bone Marrow Donors Registry (IBMDR) report published on February, 2010 [15]. This is the largest published national database of bone marrow healthy donors and includes a cohort of 370,000 individuals. The regional distribution of the HLA, A, B, C alleles was also evaluated in a more recent IBMDR high-definition-analysis database including a further 120,926-individual sample size typed with a high resolution method, which did not show significant changes compared to the previous report [16]. These data extrapolated from healthy donors represent a reliable surrogate of the real HLA-allele frequency scenario existing within the Italian population inhabiting different geographic areas of the country.

We examined allele prevalence across the Italian regions and detected a higher frequency of HLA-A*25, B*08, B*44, B*15:01, B*51, and C*01, and C*03 alleles in the northern regions compared with that recorded in the southern regions. A reverse situation was instead observed for HLA-B*14, B*18, and B*49 alleles, for which frequency is higher in the southern regions (Figure 1c,d and Supplementary Table S1). All of the other HLA A, B, and C alleles in the database did not show substantial inter- and intraregional differences (data from IBMDR database) [15].

**Figure 2:**
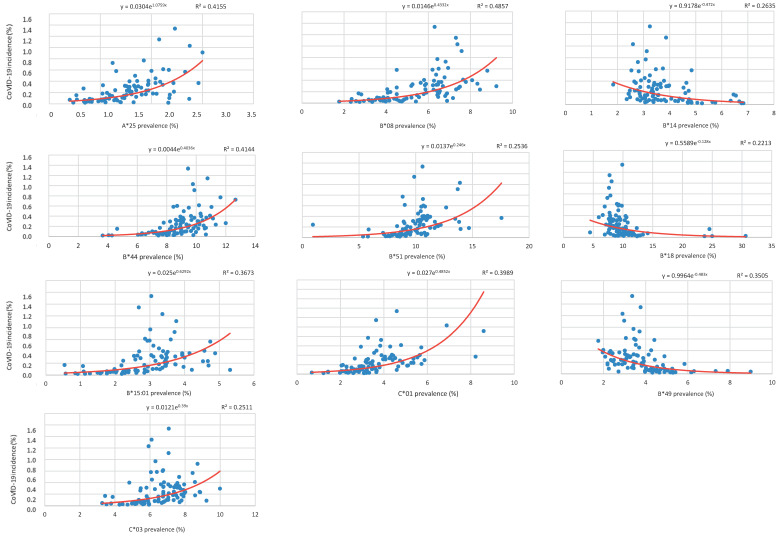
Correlation between COVID-19 incidence rate and HLA prevalence. The graphs show the correlation between COVID-19 incidence and the prevalence of HLA-A*25, B*08, B*44, B*15:01, B*51, B*14, B*18, B*49, C*01, and C*03, expressed as percentages, for all the available Italian provinces. For each correlation, the R-squared value is provided at the top of the graph along with the estimated regression equations. The r and *p* values are reported in Table 1.

We subsequently analyzed all the selected alleles (HLA-A*25, B*08, B*44, B*15:01, B*51, B*14, B*18, B*49, C*01, and C*03) and compared them with COVID-19 incidence, as reported by the Italian Department of Civil Protection. Our results showed the existing correlation between HLA allele frequency and COVID-19 incidence rate fixed on 9 April 2020 in proximity of the national outbreak peak, suggesting that the shape of the relationship is non-linear: as HLA prevalence increases, COVID-19 incidence varies according to an exponential trend (Figure 2). In particular, HLA-A*25, B*08, B*44, B*15:01, B*51, C*01, and C*03 alleles showed a positive log-linear correlation with COVID-19 incidence rate. On the other hand, HLA-B*14, B*18, and B*49 alleles, whose frequency is higher in the southern regions, showed an inverse log-linear correlation (Figure 2).

Pearson’s coefficient (r) was calculated as a measure of the correlation between the logarithm of the COVID-19 incidence and the prevalence of different HLA alleles, in accordance with the exponential model. The complete correlation matrix with Pearson’s coefficients and corresponding *p*-value for each couple of variables are shown in Table 1. HLA alleles that were positively correlated to COVID-19 infection revealed Pearson’s coefficient values between 0.50 and 0.70 (*p* < 0.0001), and those inversely correlated between −0.47 and −0.59 (*p* < 0.0001).

In order to control for mutual confounding (also including the regions in the model as confounders), the above-mentioned HLA-alleles were examined simultaneously by performing a multivariable regression analysis whose results showed that only HLA-B*44 and C*01 alleles maintained a positive and independent association with COVID-19 incidence (Table 2).

The exponential of the regression coefficient (growth rate) allowed us to quantify an increase in 16% (95%CI: 0.1–35%) in COVID-19 incidence per one percentage point increase in B*44 prevalence (Table 2). Considering that the range of B*44 prevalence varies in Italy from 4% to 12%, the risk of developing COVID-19 for the highest level of prevalence can be estimated as three times greater than that for the lowest one (Table 2). 

Similarly, for C*01 the growth rate was 19% (95%CI: 1–41%): considering a range of prevalence among provinces from 1% to 9%, the risk of developing COVID-19 for the highest level of prevalence is four times higher.

**Figure 3:**
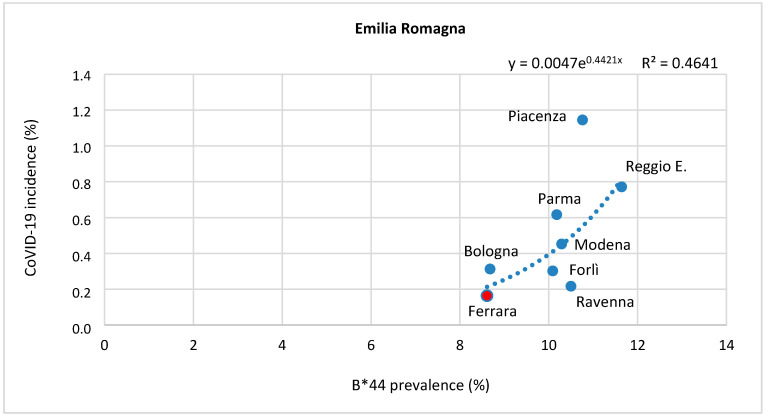

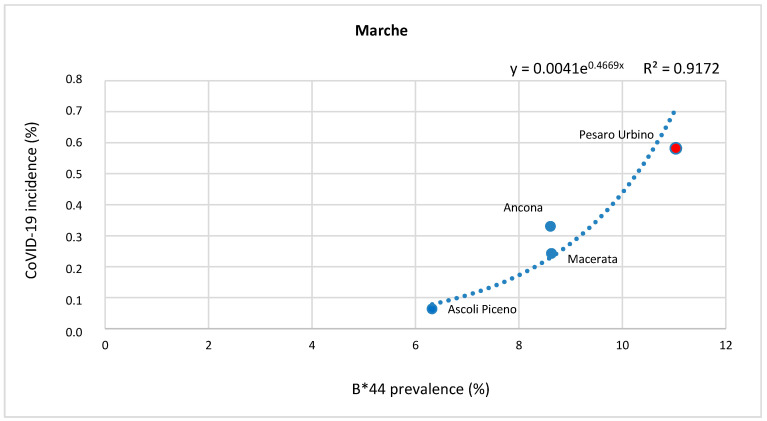
Correlation between COVID-19 incidence rate and HLA-B*44 prevalence in Emilia Romagna and Marche provinces. The graphs show the correlation between COVID-19 incidence and the prevalence of HLA-B*44 prevalence, both expressed as percentages, for all the provinces of Emilia Romagna (top panel) and all the available provinces of Marche (bottom panel). For each correlation, the R-squared value is provided at the top of the graph along with the estimated regression equations. For Emilia Romagna: r = 0.681 and *p* value = 0.0628; for Marche r = 0.958 and *p* value = 0.0423.

In order to provide further proof of evidence concerning the correlation of permissive HLA allele prevalence and COVID-19 incidence, we focused on two regions (Emilia Romagna and Marche) where the prevalence of B*44 and C*01 alleles is unevenly distributed among the different provinces (Figure 3).

Remarkably, in these regions, the identified correlation seems to account for the intra-regional differences that are currently unexplained, such as the low incidence of COVID-19 in the province of Ferrara, compared with the other Emilian provinces, which are highly affected by the virus. Similarly, Pesaro-Urbino, which is the most affected province in the Marche Region, is also one with the highest prevalence of HLA-B*44 (Figure 1e,f). In the latter case, the exponential regression curve explains almost all the variance in the data (r^2^ = 0.9172) and the prevalence of B*44 can almost exactly predict the incidence of COVID-19 (Figure 3).

## 3. Discussion

Our epidemiologic analysis, through a geographical ecological approach, identified putative permissive class I alleles that are potentially unable to trigger an efficient immune-response unable to counteract SARS-Cov-2 infection.

In particular, we selected, from the widest national genetic study that reports HLA data (in terms of allele prevalence) from almost 500,000 bone marrow donors representing the population from the whole national territory, those that presented stable inter- and intra-regional differences in prevalence, to examine whether they could underlie the geographic differences in COVID-19 incidence. By univariate analysis we found that HLA-A*25, B*08, B*44, B*15:01, B*51, and C*01, and C*03 alleles showed a positive correlation with COVID-19 incidence rate, whereas HLA-B*14, B*18, and B*49 showed an opposite trend.

Then, we tested the association between COVID-19 incidence and HLA alleles independently of each other using a multivariable regression analysis. Importantly, as an alternative approach to stratified analysis, the Italian regions were included as covariates in the model to control for the confounding effect of the geographical context, and, at the same time to verify, the association of interest regardless of the North–South gradient.

Interestingly, none of the selected alleles could be independently associated with COVID-19 incidence if not associated with the permissive HLA B*44 and C*01.

Our results are not surprising considering that class I HLA molecules have the specific task of binding and presenting antigen-derived epitope peptides to the TCR of epitope-peptide-specific T cells. With this mechanisms class I HLA molecules are critically involved in both CTL replication and ability to recognize and destroy virus-infected target cells. These 9–10 mere epitope peptides derive from the intracellular processing of protein antigens operated by the proteasome system prior being complexed with HLA molecules and translated on the membrane in order to be exposed to the TCR of the immune-effectors [17,18,19,20]. A peptide’s ability to bind HLA molecules is allele-specific and is restricted by specific amino-acidic consensus motifs that allow their anchorage to different HLA molecules [21,22]. In this context, two individuals carrying the same antigen but different HLA profile may give rise to a completely different T-cell-mediated immune-response, since they may have completely different amounts of HLA-specific antigen-derived epitopes. This hypothesis has been confirmed in several studies concerning a number of different viruses as well as tumour antigens and autoimmune models [11,23,24,25,26,27,28,29].

Our model suggests that healthy individuals carrying HLA-B*44 and/or C*01, and to a lesser extent, HLA-A*25, HLA-B*08 alleles may be more susceptible to SARS-CoV-2 infection; indeed, they could be unable to present a sufficient amount of immune-dominant virus derived epitope peptides and consequently, they would be unable to mount a fast and efficient anti-viral immune response. It can be hypothesized that, in these patients, the virus may freely spread from the oropharyngeal mucosae, starting a more efficient replication. Consistently, both HLA-B*44 and C*01 alleles, that we identified as possibly permissive to SARS-CoV-2 infection in Italy, have also been associated to known inflammatory autoimmune diseases [30,31,32,33,34], a fact that highlights their ability to trigger non-proficient and often inappropriate immunological reactions. The latter finding deserves to be explored in direct experimental approaches aimed to investigate whether the expression of these HLA alleles also correlates with more aggressive disease outcomes and the development of interstitial pneumonitis. Interestingly, inheritance of HLA-B*44 was shown to underlie susceptibility to recurrent sinopulmonary infection [35] A further consideration stems from the knowledge that the HLA-C*01 allele, which was the most permissive to SARS-CoV-2 infection in our study, also represents the specific ligand of killer cell immunoglobulin like receptors (KIRs), KIR2DL2 and KIR2DL3 [36,37,38]. These receptors are able to inhibit the activity of Natural killer cells, which represent the first line of host defence to the infection before the occurrence of a more specific T cell response [39]. This hypothesis deserves further and more accurate investigation.

In the present study, we could not assess whether a correlation existed among HLA alleles and COVID-19-associated morbidity (hospitalization) and lethality rates owing to the absence of reliable data and the presence of significant confounding factors, including a delay in hospital records transmission, the saturation of emergency rooms and the presence of co-morbidities. However, our data concerning the number of COVID-19 cases might be biased towards more severe outcomes because the initial national screening was mostly limited to symptomatic or hospitalized individuals, therefore implying in our correlation analyses an association with severity of the disease.

Although this type of ecological approach has intrinsic limits, it also has the advantage of considering a large number of cases which are readily available through public-access datasets. Indeed, geographical ecological studies are often the first to identify risk factors for a variety of diseases, which are then verified through subsequent studies [40]. 

Our observational study identifies HLA-C*01 and B*44 alleles as potential genetic determinants for the identification of individuals at risk, which warrants further investigation in case-control studies. To this purpose, we are currently investigating the expression of different HLA alleles in pauci-symptomatic patients affected by COVID-19 and those with severe interstitial pneumonitis. We suppose that Class I and II HLA genotyping in COVID-19 patients could be easily achieved and cost-effective and could provide the basis for the identification of individuals with a high risk of interstitial pneumonitis and cytokines’ storm who should be immunised first when a vaccine becomes available.

Overall our results, by identifying the potential relevance of HLA-C*01 and B*44 alleles in developing COVID-19, open new avenues of investigation, not only to understand the diffusion and the physio-pathogenesis of the disease, but also to inform future vaccination campaign priorities and clinical management strategies while promoting the research of other potential permissive alleles and high-risk population worldwide.

## 4. Materials and Methods 

### 4.1. Data source and Population Sample

HLA is a highly polymorphic genetic system. The frequency of specific HLA alleles varies significantly among the different populations inhabiting the twenty geographic regions that compose the Italian Republic. We retrieved the HLA allele frequency data, which were recorded within the different regions and relative intra-regional provinces, from the database of the IBMDR. We referred to an IBMDR database analysis, published on 1 February 2010 [15], containing data that were collected in a twenty year interval from a cohort of 370,000 volunteer donors with known provincial and regional birthplace origin. These allele frequencies, expressed in % as calculated through the Arlequin Software, were also compared to those reported in an updated version of the IBMDR database, including data collected on a further 120,926 volunteer donor cohort [16]. Samples from the more recent cohort were typed with a high-resolution method [16]; frequency data were grouped in regions and the same allele nomenclature version was used (according to the Immuno Polymorphism Database(IPD)-International ImMunoGeneTics project (IMGT/HLA) Database Release 3.32, April 2018; http://www.ebi.ac.uk/ipd/imgt/hla/stats.html; (access on 14 July 2020). The same allele frequency distribution across Italy was confirmed.

Data concerning the total number of individuals infected by SARS-CoV-2 per province (updated to 9 April 2020) were provided by the Italian Department of Civil Protection, the institution under the Presidency of the Council of Ministers that manages the emergency at national level. Data were provided as aggregate numbers, in an anonymized manner.

### 4.2. Statistical Analysis

The relationship between SARS-CoV-2 infection and the frequency of HLA alleles was explored as part of an ecological approach aimed at assessing the degree of correlation between the incidence of COVID-19 and the prevalence of HLA alleles, both measured on a geographical basis (taking each Italian province as the unit of observation).

For each allele, values were preliminarily plotted in a scatter diagram and the curve with the best fit (corresponding to exponential curve) was selected using the least squares method, which is the most widely used procedure for developing estimates of the model parameters. The estimated regression equations are indicated at the top of each graph (Figure 2 and Figure 3).

Consistent with the exponential model, Pearson’s coefficient (r) was calculated as a measure of the correlation between the logarithm of the COVID-19 incidence and the prevalence of different HLA alleles. For each value of r, the corresponding *p*-value was also considered, in order to assess the statistical significance of the correlation (with respect to the null hypothesis of no log-linear correlation).

Finally, the association between the logarithm of the COVID-19 incidence (considered as dependent variable) and HLA alleles independently of each other was tested using a multivariable regression analysis. Furthermore, the Italian regions were included as covariates in the model (which is an alternative approach to stratified analysis), in order to control for the confounding of geographical context and, at the same time, to verify the association of interest regardless of the North–South gradient.

All statistical analyses were conducted using STATA software 11.0 version (StataCorp LLC, College Station, Texas, TX 77845-4512, USA). Microsoft Excel was used to draw maps.

## Figures and Tables

**Table 1:** – Matrix of correlation (Pearson’s coefficient and *p*-value) between COVID-19 incidence rate and HLA.

	COVID-19	A*25	B*08	B*14	B*18	B*44	B*49	B*51	B*15:01	C*01	C*03
**COVID-19 †**	1.0000										
A*25	0.6446	1.0000									
	*p* < 0.0001										
B*08	0.6969	0.7196	1.0000								
	*p* < 0.0001	*p* < 0.0001									
B*14	−0.5133	−0.4193	−0.5617	1.0000							
	*p* < 0.0001	*p* < 0.0001	*p* < 0.0001								
B*18	−0.4704	−0.4573	−0.6161	0.6053	1.0000						
	*p* < 0.0001	*p* < 0.0001	*p* < 0.0001	*p* < 0.0001							
B*44	0.6438	0.5555	0.6865	−0.5512	−0.7056	1.0000					
	*p* < 0.0001	*p* < 0.0001	*p* < 0.0001	*p* < 0.0001	*p* < 0.0001						
B*49	−0.5920	−0.6280	−0.7144	0.5331	0.3019	−0.5715	1.0000				
	*p* < 0.0001	*p* < 0.0001	*p* < 0.0001	*p* < 0.0001	*p* = 0.0033	*p* < 0.0001					
B*51	0.5036	0.5478	0.6196	−0.4405	−0.4851	0.4296	−0.5702	1.0000			
	*p* < 0.0001	*p* < 0.0001	*p* < 0.0001	*p* < 0.0001	*p* < 0.0001	*p* < 0.0001	*p* < 0.0001				
B*15:01	0.6060	0.5780	0.6826	−0.6238	−0.5760	0.6092	−0.6247	0.5695	1.0000		
	*p* < 0.0001	*p* < 0.0001	*p* < 0.0001	*p* < 0.0001	*p* < 0.0001	*p* < 0.0001	*p* < 0.0001	*p* < 0.0001			
C*01	0.6316	0.6367	0.6196	−0.3433	−0.2754	0.3501	−0.6037	0.6752	0.4997	1.0000	
	*p* < 0.0001	*p* < 0.0001	*p* < 0.0001	*p* = 0.0008	*p* = 0.0075	*p* = 0.0006	*p* < 0.0001	*p* < 0.0001	*p* < 0.0001		
C*03	0.5011	0.4817	0.5527	−0.5509	−0.5378	0.4638	−0.5607	0.4817	0.7834	0.4396	1.0000
	*p* < 0.0001	*p* < 0.0001	*p* < 0.0001	*p* < 0.0001	*p* < 0.0001	*p* < 0.0001	*p* < 0.0001	*p* < 0.0001	*p* < 0.0001	*p* < 0.0001	

(†) incidence rate (at logarithm base).

**Table 2:** – Multiple regression model: COVID-19 incidence rate and HLA.

COVID-19	Regression Coefficient	Adjusted Growth Rate †	(95% CI)	*p*-Value
A*25	0.2908	1.34	(0.86–2.08)	n.s
B*08	0.0804	1.08	(0.90–1.30)	n.s
B*14	0.0805	1.08	(0.88–1.33)	n.s
B*18	0.0492	1.05	(0.94–1.17)	n.s
B*44	0.1484	1.16	(1.00–1.35)	0.050
B*49	0.1431	1.15	(0.93–1.43)	n.s
B*51	−0.0174	0.98	(0.89–1.08)	n.s
B*15:01	−0.0305	0.97	(0.73–1.29)	n.s
C*01	0.1747	1.19	(1.01–1.41)	0.042
C*03	−0.0530	0.95	(0.78–1.15)	n.s

† also adjusted for region (using a multiple regression model).

## Supplementary Materials

Supplementary materials can be found at https://www.mdpi.com/1422-0067/21/15/5205/s1. Table S1. HLA AND COVID-19 data Italy.
